# Brief instruments for measuring nutrition literacy - the Nutrition Health Literacy Scale and the Self-Perceived Food Literacy Scale Short Form

**DOI:** 10.1186/s12937-024-00971-z

**Published:** 2024-07-11

**Authors:** Robert Griebler, Denise Schütze, Thomas Link, Karin Schindler

**Affiliations:** 1 Austrian National Public Health Institute, Stubenring 6, Vienna, 1010 Austria; 2 Department of Internal Medicine III, Div. Endocrinology and Metabolism, Medical University Vienna, Währinger Gürtel 18-20, Vienna, 1090 Austria

**Keywords:** Health literacy, Nutrition health literacy, Nutrition literacy, Food literacy, Measure, Validity

## Abstract

**Background:**

A healthy diet is a critical factor in maintaining long-term health. In addition to a health-promoting food environment, the nutrition health literacy (NHL) and food literacy (FL) of the population are important in this context. This paper describes the development and validation of two short instruments to measure the nutrition literacy of the population, used in the Austrian Nutrition Literacy Survey 2021.

**Methods:**

An instrument to measure NHL (Nutrition Health Literacy Scale; NHLS) has been adapted and further developed. To measure FL, the Self-perceived Food Literacy Scale by Poelman et al. has been modified and shortened (SPFL-SF). Validation of the instruments was based on data from a web survey conducted in Austria in 2021 with almost 3,000 participants aged 18 years and older. Exploratory and confirmatory factor analyses were performed to assess the factorial validity/dimensionality of the instruments. Additionally, internal consistency was assessed using Cronbach’s alpha, ordinal alpha, and McDonald’s omega.

**Results:**

Both instruments demonstrate excellent data-model fit. The NHLS also shows excellent internal consistency (α = 0.91), while the SPFL-SF displays a sufficient internal consistency for all (α between 0.70 and 0.89) but one sub-dimension (resisting temptation α = 0.61). Furthermore, the distribution of the items indicates that the measures are understandable and suitable, as evidenced by the absence of missing values in the sample. In addition, the items of both instruments differ in their level of difficulty or agreement.

**Conclusions:**

The NHLS and SPFL-SF are reliable and valid instruments for measuring NHL and FL in the general adult population. The brief instruments measuring the different aspects of nutrition literacy can be easily used in nutritional or evaluation studies. Further work is required to investigate other aspects of validity.

## Background

The importance of nutrition for health, particularly in the prevention of obesity and non-communicable diseases, is undisputed [1]. In recent years, however, the impact of nutrition behaviour and food systems on the environment and climate change has become increasingly recognized. This makes it even more important that consumers have access to a wide range of healthy and sustainable food and are motivated and able to make healthy but also informed and sustainable choices. In this context, food literacy (FL) and nutrition health literacy (NHL) are considered as crucial to maintain a healthy and sustainable diet.

This is even more important, as many countries worldwide experience a high prevalence of persons with overweight [2, 3]. The Austrian Nutrition Survey measured not only body weight and height, but also nutrient intake and the intake of foods from the various food groups. The survey shows that Austrians consume an unbalanced diet with too many foods of animal origin, too many foods high in sugar and not enough vegetables and fruits [4]. This picture did not change much over the years [5]. Major factors contributing to health promoting dietary habits are NHL and FL [6, 7].

NHL is understood as a subcategory of health literacy. It refers to the extent to which individuals are able to access, understand, appraise, and apply nutrition information needed to make appropriate everyday nutrition decisions for better health and well-being [8–10]. This understanding follows the general definition of HL as presented by the HLS-EU consortium [11]. Understood as a relational concept [12], NHL does not only emerge from personal motivation and capabilities but in conjunction with the availability, accessibility, comprehensibility, quality, and user-friendliness of nutrition-related information and services.

The term FL refers to the competences and practices of healthy eating [13]. Compared to NHL, FL is a broader concept, encompasses a collection of interrelated knowledge, skills, and behaviours, and extends to other determinants that may influence food decisions, such as social and cultural factors [10]. In the scientific literature, the FL concept is defined more narrowly or more broadly, depending on how it is formulated [14].

During the last years, the Austrian Ministry of Health and the administration of the federal regions have developed nationwide and regional (nutrition) health promoting initiatives. To ensure the success of nutrition health promoting initiatives and to make them even more successful, initiatives should take peoples’ food and nutrition competencies and motivation into account. Current measures for NHL focus primarily on a functional understanding of NHL (reading skills, comprehension of nutritional information), while those for FL are often lengthy and time-consuming [15, 16]. To facilitate a targeted approach, we have further developed and validated two instruments that can be used in nutrition and evaluation studies: an adapted and further developed short instrument on NHL and a modified and shortened instrument on FL [8].

## Materials and methods

### Nutrition Health Literacy Scale (NHLS)

An instrument to measure NHL in adults (Nutrition Health Literacy Scale; NHLS) has been adapted and further developed. The NHLS was first developed as part of a project to measure HL in children and adolescents (NHLS-C) [17]. It has been modified and extended for the use in adult populations. Based on Sørensen’s concept of HL [11], the NHLS measures self-rated difficulties in accessing, understanding, appraising, and applying nutrition-related information for better health and well-being. The measurement of difficulty considers the relational nature of HL, as difficulties may arise from a low personal competence in processing information and/or from a challenging information environment (e.g., in terms of availability, comprehensibility, quality, and user-friendliness, etc.). Respondents rate the difficulty of specific NHL tasks on a 5-point Likert scale (1 very difficult − 2 rather difficult − 3 neither/nor − 4 rather easy – 5 very easy). A total of 16 items were used to measure NHL (4 items on accessing, 3 items on understanding, 5 items on appraising, and 4 items on applying nutrition-related information). The item set originally developed in German, was validated with nutrition experts, and shortened to 12 items, three for each process dimension, as part of the analyses.

### Self-Perceived Food Literacy Scale Short Form (SPFL-SF)

The Self-Perceived Food Literacy Scale (SPFL), originally developed by a Dutch research team [18], measures individuals’ self-rated competencies and practices related to healthy eating, based on 29 items covering various aspects of FL. The items are to be answered on a 5-point Likert scale (1 no, never – 2 no, usually not – 3 sometimes yes, sometimes no – 4 yes, mostly – 5 yes, always). Three items are reverse coded. The German version of the instrument was provided by the authors of a German study [19]. This version was linguistically adapted to the Austrian context, modified in two places, and extended by one question. As part of the analyses, the instrument was shortened to a 20 items short form (SPFL-SF).

### Data collection

The assessment of the psychometric properties of the questionnaires is based on data from the Austrian Nutrition Literacy Survey 2021 [8]. Respondents were recruited from the online panel of the Austrian Gallup Institute, using a stratified random sample. In rare cases, the Austrian Gallup Institute also purchased participants from other panels to obtain sufficient cases for specific population groups in small federal states (e.g., Burgenland). For stratification, the following characteristics were used: age, gender, formal education, employment status, federal regions, and degree of urbanization. The Austrian Nutrition Literacy Survey 2021 was conducted between October 11 and November 8, 2021. The questionnaire was previously field tested (*n* = 48). In this context, the comprehensibility of the questions, technical deficiencies in the programming of the questionnaire, and the duration of the survey were examined. The field test did not reveal any problems.

Deviations in the composition of the sample from the population were adjusted by weighting for age, sex, formal education, employment status, federal regions, and degree of urbanization. Weighting was performed using the Random Iterative Method (RIM) with weights ranging from 0.173 to 5.608. Table 1 shows the distribution of the target population and the distribution of the unweighted sample and the weighted data according to the variables mentioned. The weighted data are broadly representative of the web-savvy adult population in Austria.

### Participants

The total sample consists of 2,993 adults aged 18 years and over. 51% are women. The mean and median age are 48.3 (± 16.5) and 48.0 years, respectively. In terms of educational attainment, most participants have completed intermediate vocational education or vocational training; in terms of employment status, most participants are employed; and in terms of level of urbanisation, most participants live in a city (see Table 1).

**Table 1:** Participants by socio-demographic factors (unweighted and weighted)

Characteristics	n (%) target population; *n* = 7,125,314	n (%) unweighted; *n* = 2993	n (%) weighted; *n* = 2993
**Gender**			
Male	3,477,153 (49)	1452 (49)	1458 (49)
Female	3,648,161 (51)	1537 (51)	1532 (51)
Diverse gender^a^	-	4 (0)	3 (0)
**Age**			
18–34 years	1,895,334 (20)	783 (21)	796 (20)
35–49 years	1,816,955 (21)	779 (2)	763 (21)
50–64 years	1,845,456 (21)	801 (20)	775 (21)
65 years or more	1,567,569 (22)	630 (23)	658 (22)
**Education**			
Compulsory education	1,389,436 (24)	239 (8)	584 (24)
Intermediate vocational education/vocational training	3,462,903 (49)	1548 (52)	1454 (49)
A-level or higher	2,272,975 (32)	1206 (40)	956 (32)
**Employment status**			
Employed	3,833,418 (54)	1637 (55)	1599 (53)
Unemployed	206,634 (3)	151 (5)	184 (6)
Non-employed^b^	3,085,261 (43)	1205 (40)	1211 (41)
**Level of urbanisation**			
City	2,237,348 (31)	1266 (42)	1278 (43)
Small town/suburb	2,194,597 (31)	634 (23)	613 (23)
Rural area	2,693,369 (38)	1093 (37)	1103 (37)

Source of target population: Statistics Austria (Microcensus 2017) (24).

^a^ People who reported being diverse were not included in the analyses due to their small numbers

^b^ Retired, unable to work for health reasons, student, purely fulfilling domestic tasks, in compulsory military or civilian service

### Analysis

To test the *factorial validity/dimensionality* of the new NHLS and the slightly adapted SPFL, we conducted exploratory factor analyses (EFA) to examine the factor structure of the instruments and confirmatory factor analyses (CFA) to test whether the data fit the theoretically hypothesised factor model.

Principal axis factoring (PAF) was used as the EFA extraction method. Oblimin with Kaiser normalisation was chosen as the rotation method. Factor loadings should be greater than or equal to 0.5 [23].

For the CFA of ordinal data, DWLS (Diagonally Weighted Least Squares) estimates were calculated [22]. Model fit was determined using Standardized Root Mean Square Residual (SRMSR, ≤ 0.08), Root Mean Square Error of Approximation (RMSEA, ≤ 0.06), Tucker-Lewis Index (TLI, ≥ 0.95), and Comparative Fit Index (CFI, ≥ 0.95). Thresholds from Prudon [25] and Beaujean [26] were applied. Furthermore, correlation residuals should not exceed 0.2 [22].

The *internal consistency* of the instruments was assessed using Cronbach’s Alpha, Ordinal Alpha, and McDonald’s Omega. For all parameters, a value of 0.70 or higher is considered acceptable [21, 27]. Following the recommendation by Fornell & Larcker [20], the average variance extracted (AVE) was calculated. The AVE should be 0.5 or greater so that the amount of variation captured by the measurement instrument is not smaller than the variance due to measurement error.

The mean, standard deviation, median, percentiles, skewness and kurtosis are calculated to describe the distribution of the NHLS and the SPFL-SF score.

Analyses were performed on the weighted data using SPSS or the R package lavaan1 [28].

## Results

### Exploratory factor analysis

Examining the item set of the adapted and further developed NHLS, four dimensions reflecting accessing, understanding, appraising, and applying nutrition-related information were found in the EFA. To balance the number of items per dimensions represented, the instrument was shortened to 12 items (three items per dimension).

The factor structure of the slightly adapted SPFL, consisting of 29 + 1 items, revealed ten dimensions representing different aspects of FL. This is two more dimensions than those reported by Poelman et al. [18], with the two additional dimensions further differentiating two original dimensions: The dimension “food preparation skills” is subdivided into “healthy cooking” and “assessing food quality”, the dimension “resilience and resistance” is subdivided into “resisting temptations” and “healthy eating in exceptional situations”. Each of the dimension consists of a different number of items and therefore has a different impact on the overall SPFL score. To obtain a shorter, more concise, and balanced instrument, a short version (SPFL-SF) with only two items per dimension was developed based on content and statistical considerations in consultation with nutrition experts.

### Confirmative factor analysis

**Fig. 1:**
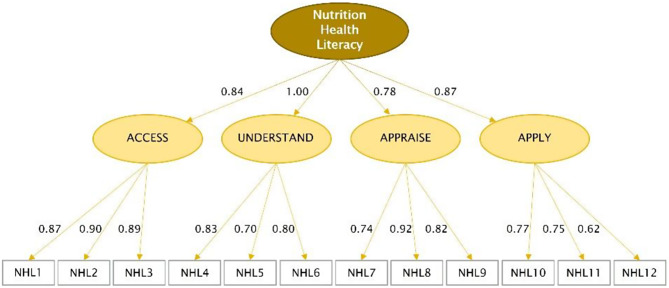
Confirmatory factor analysis of the NHLS as a hierarchical model with four sub-dimensions, factor loadings (weighted data, *n* = 2993)

A test of the factor structure of the NHLS based on the original 16 items (corresponding to the four sub-dimensions and considering a hierarchical factor structure) did not result in a satisfactory data model fit. To obtain a shorter and balanced version, the item set was reduced to 12 items (three items per sub-dimension) based on content and statistical considerations in consultation with nutrition experts. The reduced item set (see Fig. 1) provided an excellent fit to the model (Table 2). All factor loadings are above a value of 0.5 and no correlations of residuals > 0.2 were found.

**Fig. 2:**
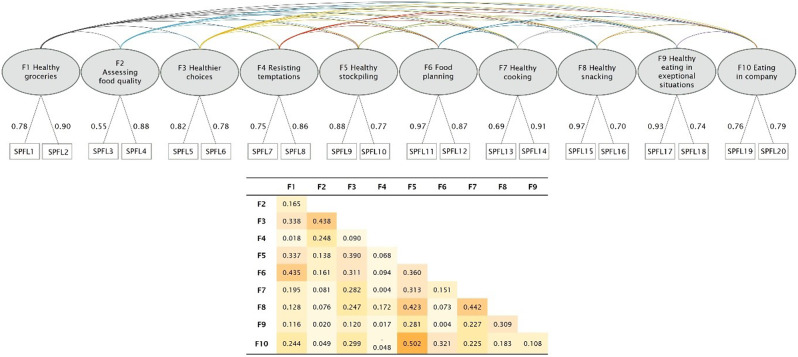
Confirmatory factor analysis of the SPFL-SF as a 10-factor model, factor loadings (weighted data, *n* = 2993)

Testing of the 20-item SPFL-SF shows an excellent fit of the data to the hypothesized 10-factor model (Table 2). All factor loadings are above a value of 0.5 and no correlations of residuals > 0.2 were found. The SPFL-SF is highly correlated with the long form of the SPFL (*r* = 0.942, *p* < 0.001). (Fig. 2) 

**Table 2:** Psychometric properties of the NHLS and SPFL-SF (weighted data, *n* = 2993)

Parameters	NHLS (hierarchical 4-factor model)	SPFL-SF (10-factor model)
Cronbach’s alpha	0.91	0.77
Cronbach’s alpha subdimensions	0.71–0.87	0.61–0.89
Ordinal alpha	0.76–0.91	0.65–0.92
McDonald’s Omega	0.71–0.88	0.66–0.89
Average variance extracted (AVE)	0.51–0.78	0.54–0.85
Standardized Root Mean Square Residual (SRMSR)	0.03	0.02
Root Mean Square Error of Approximation (RMSEA)	0.05 (CI 0.05–0.06)	0.02 (CI 0.02–0.02)
RMSEA p-value	0.15	1.00
Tucker-Lewis Index	1.00	1.00
Comparative Fit Index (CFI)	1.00	1.00
Correlation residuals > 0.2	none	none

### Reliability

A Cronbach’s alpha of 0.91 indicates excellent internal consistency for the NHL scale. The lowest Cronbach’s alpha value for the subdimensions of the NHLS is still acceptable at 0.71.

The Cronbach’s alpha of the SPFL-SF is sufficient with 0.77. However, one sub-dimension is below the threshold (resisting temptation; α = 0.61), but all sub-dimensions consist of only two items.

Similar results were found for the ordinal alpha, McDonald’s Omega and in relation to the AVE (see Table 2).

### Distribution of the NHLS and SPFL-SF items

Table 3 provides information on the distribution of the 12 NHLS items (mean, standard deviation (SD), and proportion of respondents answering the extreme categories “very difficult” and “very easy”. Five NHLS items were rated as “very easy” by about one-fifth to one-quarter of respondents (NHL1, NHL2, NHL3, NHL4, and NHL10), indicating that these items are easy to endorse. For all other items the value is below 16%. Three of the five items represent the sub-dimension “accessing”.

The items vary in difficulty, with the percentage of “very difficult” ratings ranging from 2 to 11%, and “very easy” ratings ranging from 8 to 24%.

There were no missing values, indicating that the items were understandable and suitable.

**Table 3:** Descriptive results for the NHLS items (weighted data, *n* = 2993)

Dimension	Item number	It is not always easy to get understandable, reliable, and useful information about nutrition. With the following questions, we would like to find out what challenges exist when dealing with information about healthy eating. On a scale from easy to difficult, how easy would you say it is for you to …	Mean (± SD)	Percentage of “very difficult” and “very easy”	
Access	NHL1	find or get useful information about which foods are healthy and which are less healthy?	3.7 (± 1.0)	2	21
Access	NHL2	find or get useful information about what you should eat and drink more or less of to maintain a healthy diet?	3.7 (± 1.0)	3	21
Access	NHL3	find out what to look for in a healthy diet?	3.8 (± 1.0)	2	22
Understand	NHL4	understand information about why some foods are healthy and others are not?	3.8 (± 1.0)	2	24
Understand	NHL5	understand the information about ingredients on food packaging (list of ingredients and nutritional table)?	3.2 (± 1.2)	7	15
Understand	NHL6	understand dietary recommendations that tell you how often, what, and how much you should eat and drink to maintain a healthy diet?	3.5 (± 1.0)	3	16
Appraise	NHL7	judge whether you can trust food advertising?	3.0 (± 1.2)	11	12
Appraise	NHL8	judge whether information about healthy eating is right or wrong?	3.1 (± 1.0)	5	9
Appraise	NHL9	judge how trustworthy information about healthy eating is on the internet and social media?	3.0 (± 1.1)	10	8
Apply	NHL10	decide what to eat and drink more or less of in order to maintain a healthy diet?	3.7 (± 1.0)	2	20
Apply	NHL11	explain to others in an understandable manner what a healthy diet is all about?	3.3 (± 1.1)	6	12
Apply	NHL12	follow recommendations for a healthy diet?	3.2 (± 1.1)	6	10

*Note* The English translation of the NHLS was produced in collaboration with the SHIFT2HEALTH project (https://shift2health.eu/what-is-shift2health/), following the full standard translation process

Table 4 shows information on the distribution of the 20 SPFL-SF items (mean, standard deviation (SD), and proportion of respondents answering the extreme categories “no, never” and “yes, always”. Eight of the SPFL-SF items were rated either as “no, never” (SPFL9 and SPFL14) or “yes, always” (SPFL1, SPFL2, SPFL4, SPFL13, SPFL19, and SPFL20). For ten items the value is ≤ 15%. Six of the eight items represent the sub-dimensions “healthy groceries”, “healthy cooking”, and “eating in company”.

The responses to the SPFL-SF items vary in their level of agreement, with “no, never” responses ranging from 1 to 29%, and “yes, always” responses ranging from 5 to 52%.

Again, the lack of missing values indicates that the SPFL-SF items were understandable and suitable.

**Table 4:** Descriptive results for the SPFL-SF items (weighted data, *n* = 2993)

Dimension	Item number	Question:	Mean (± SD)	Percentage of “no, never” and “yes, always”	
Healthy groceries	SPFL1^a^	Do you buy healthy foods, such as vegetables, fruit, or whole grain products?	4.2 (± 0.8)	1	38
	SPFL2	Do you purchase healthy foods, even if they are a bit more expensive? *For example, vegetables, fruit, or whole grain products?*	3.8 (± 0.9)	2	23
Assessing food quality	SPFL3^b^	Can you recognise the quality of meat/fish by sight or smell?	3.6 (± 1.1)	4	17
	SPFL4^b^	Can you recognise the quality of fruit/vegetables by sight or smell?	4.0 (± 0.8)	1	27
Healthier choices	SPFL5	Do you compare the calories, fat, sugar or salt content of different products?	2.6 (± 1.2)	19	6
	SPFL6	Do you check the nutritional labels of products for calories, fat, sugar, or salt content?	2.9 (± 1.1)	13	8
Resisting temptations	SPFL7	Imagine that you are at a place where you see and smell tasty foods. Are you able to resist the temptation of buying them? *For example, at the train station, petrol station, or bakery?*	3.4 (± 0.9)	1	13
	SPFL8	Are you able to say ‘no’ to tasty snacks if you want to? *For example, birthday treats or finger food?*	3.4 (± 1.0)	3	12
Healthy stockpiling	SPFL9^r^	Do you have 4 or more packages of crisps, pretzels, or savoury snacks in stock?	2.5 (± 1.2)	23	7
	SPFL10^r^	Do you have 4 or more packages of candy, cookies, or chocolate in stock?	2.8 (± 1.2)	15	11
Food planning	SPFL11	If you have something to eat, do you take account of what you will eat later that day?	3.3 (± 1.0)	5	12
	SPFL12	If you have something to eat, do you reflect on what you have eaten earlier that day?	3.3 (± 1.1)	5	12
Healthy cooking	SPFL13	Are you able to prepare a meal using fresh ingredients? *So, without pre-packed and processed foods?*	4.3 (± 0.9)	1	52
	SPFL14^r^	Do you find it difficult to prepare a meal with more than five fresh ingredients?	2.2 (± 1.1)	29	5
Healthy snacking	SPFL15	Do you eat vegetables as snacks?	2.8 (± 1.0)	9	5
	SPFL16	Do you eat fruit as a snack?	3.4 (± 0.9)	3	10
Healthy eating in exceptional situations	SPFL17	Are you able to eat healthily when you feel stressed?	3.1 (± 1.0)	5	8
	SPFL18	Are you able to eat healthily if the situation deviates from a regular situation? *For example, when you have unexpected guests or experience time pressure?*	3.2 (± 0.9)	2	6
Eating in company	SPFL19	Do you find it important to eat at the dinner table if you are eating with others?	4.2 (± 1.0)	2	50
	SPFL20	Do you find it important to eat dinner at the same time if you are with others?	4.1 (± 1.0)	2	41

*Note* The English version of the SPFL-SF is based on Poelman et al. (18) and has been adapted for items SPFL1, SPFL3 and SPFL4

^a ^Item was slightly adjusted (originally: Do you purchase healthy food, even if you have limited money?)

^b^ Items were originally one question (Are you able to see, smell or feel the quality of fresh foods? For example, of meat, fish, or fruit?)

^r ^These items need to be revised before score calculation

### Distribution of the NHLS and SPFL-SF score

The NHLS and SPFL-SF scores and their sub-scores are calculated as additive sum values, scaled from 0 to 100. To calculate SPFL-SF scores and sub-scores, items SPFL9, SPFL10 and SPFL14 must be reverse coded (5 = 1, 4 = 2, 3 = 3, 2 = 4, 1 = 5). Scores are calculated only if all items are answered validly. For all scores, a higher value indicates better results.

**Fig. 3:**
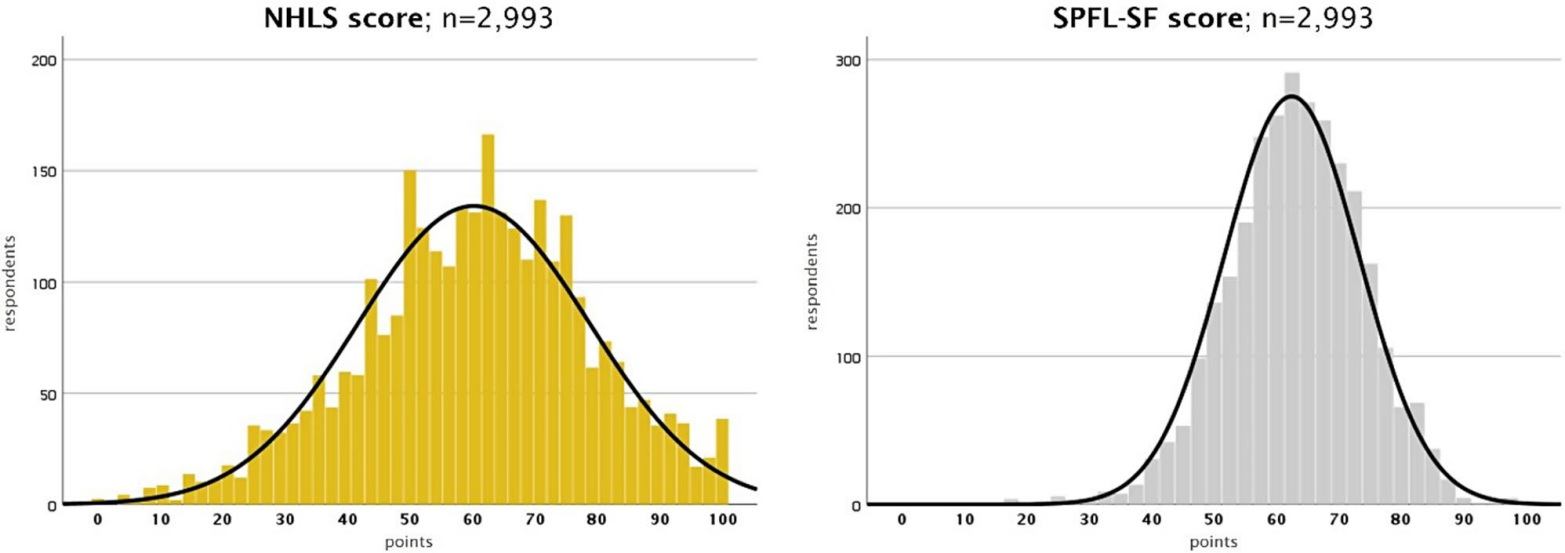
Histograms of the NHLS and SPFL-SF score (weighted data)

The mean and median of the NHLS are around 60 points, with a SD of 18.5 points (see Table 5). The distribution of the NHLS score is slightly left-skewed and mesokurtic. The mean scores of the four sub-dimensions, accessing, understanding, appraising, and applying, range from about 51 and 68 points. (Fig. 3)

The mean and median SPFL-SF score are approximately 62 points with a SD of 10.8 points. The distribution is slightly left-skewed and leptokurtic (see Table 5). The mean scores of the ten sub-dimensions range from about 44 and 79 points.

**Table 5:** Descriptive results for the NHLS and SPFL-SF score (range: 0-100, weighted data)

	NHLS score	SPFL-SF score
Mean (± SD)	60.2 (± 18.5)	62.4 (± 10.8)
Median	60.4	62.5
Minimum	0.0	15.0
Maximum	100.0	97.5
25th percentile	47.9	55.0
75th percentile	72.9	70.0
Skewness	-0.243	-0.287
Kurtosis	-0.100	0.569

## Discussion

The aim of the study was to validate two short instruments, one measuring NHL and one measuring FL. Our results indicate that the reduced item set of the adapted and further developed NHLS shows an excellent data-model fit as well as an excellent internal consistency. The same is true for the SPFL-SF, which also showed an excellent data-model fit and sufficient internal consistency for all but one sub-dimension. The results confirm the findings of Poelman et al. [2018], with two additional dimensions that further differentiate two of the original dimensions of FL. As the ten sub-dimensions cannot be hierarchically assigned to a factor, the calculation of the SPFL-SF score follows a formative logic [29].

Both instruments are balanced as the dimensions consist of the same number of items. In addition, the measures are understandable and appropriate, as evidenced by the absence of missing values in the sample. In both cases, the items vary in their difficulty or level of agreement.

As shown in a systematic review [16], instruments for measuring nutrition literacy predominantly focus on a functional understanding, such as reading skills and comprehension of nutritional information. The NHLS, however, is based on a more comprehensive public health-oriented understanding of NHL [8].

Existing measures of FL [15] often refer to the definition of FL by Vidgen and Gallegos [13], but in most cases are quite lengthy. In contrast, the SPFL-SF provides a more concise way to measure FL in its facets.

### Limitations

The sample used to validate the two instruments (NHLS and SPFL-SF) is based on an online survey. Therefore, not all population groups were reached equally well (e.g., elderly people). Furthermore, the survey was conducted in German only. The sample is therefore representative of the web-savvy, German-speaking adult population in Austria. It should also be noted that these are self-assessment tools.

## Conclusions

Both instruments (NHLS and SPFL-SF) have excellent factorial validity and sufficient internal consistency. The short instruments have been conceptually developed and validated with nutrition experts. Because of their brief nature, they are particularly suitable for measuring different aspects of nutrition literacy in nutrition studies or evaluation studies. Other aspects of validity need to be investigated in further studies.

### Use of the instruments

The NHLS and SPFL-SF have been developed by Gesundheit Österreich GmbH (GÖG). They can be used free of charge by third parties for research purposes but require a contractual agreement between the user and GÖG. Please contact the corresponding author.

## Data Availability

The dataset used in the current study is available from the corresponding author upon reasonable request.

## Abbreviations

FL: Food Literacy
HL: Health Literacy
NHL: Nutrition Health Literacy
NHLS: Nutrition Health Literacy Scale
SPFL: Self Perceived Food Literacy Scale
SPFL-SF: Self Perceived Food Literacy Scale – Short Form
